# Health system costs of providing outpatient care for diabetes in people with TB in the Philippines

**DOI:** 10.5588/ijtldopen.23.0554

**Published:** 2024-03-01

**Authors:** T. Yamanaka, M.C. Castro, J.P. Ferrer, J.A. Solon, S.E. Cox, Y.V. Laurence, A. Vassall

**Affiliations:** ^1^ Department of Global Health and Development, London School of Hygiene & Tropical Medicine (LSHTM), London, UK;; ^2^ School of Tropical Medicine and Global Health, Nagasaki University, Nagasaki, Japan;; ^3^ Nutrition Center of the Philippines, Muntinlupa City, The Philippines;; ^4^ Faculty of Epidemiology and Population Health, LSHTM, London, UK;; ^5^ Institute of Tropical Medicine, Nagasaki University (NEKKEN), Nagasaki, Japan;; ^6^ UK Health Security Agency, London,; ^7^ Health Economics for Life Sciences and Medicine, Department of Population Health Sciences, King’s College London, London, UK

**Keywords:** tuberculosis, DM, provider costs, health system costs

## Abstract

**BACKGROUND:**

Diabetes mellitus (DM) is a known risk factor for active TB. A key activity in the Philippines is to integrate TB services with other disease programmes, with a target of DM screening in 90% of TB cases. However, costs of providing DM outpatient services for TB patients are not well known.

**METHODS:**

We estimated the costs of providing integrated DM outpatient services within TB services from the health system perspective. Resources for outpatient DM services were valued using the bottom-up approach for capital goods, staff time and consumables. Resource quantities were obtained by interviewing 60 healthcare professionals in 11 health facilities in the Philippines.

**RESULTS:**

The mean cost per service ranged from USD0.53 for DM risk assessment to USD23.72 for oral glucose tolerance test. The cost per case detected for different algorithms varied from USD17.43 to USD80.81. The monthly cost per patient was estimated at USD8.95 to USD12.36.

**CONCLUSION:**

Our study provides the first estimates of costs for providing integrated DM outpatient services and TB care in a low- and middle-income country. The costs of DM detection in TB patients suggests that it may be useful to further investigate the cost-effectiveness and affordability of service delivery.

## RÉSUMÉ

**CONTEXTE:**

Le diabète (DM) est un facteur de risque bien établi pour la TB active. Aux Philippines, l'une des principales initiatives est d'intégrer les services de lutte contre la TB dans d'autres programmes de santé, dans le but de dépister le DM chez 90% des patients atteints de TB. Cependant, les coûts des services ambulatoires de traitement du DM pour les patients atteints de TB ne sont pas clairement définis.

**MÉTHODES:**

Nous avons évalué les coûts des services ambulatoires intégrés pour le traitement du DM dans le cadre des services de lutte contre la TB, du point de vue du système de santé. Les ressources pour les services ambulatoires de DM ont été évaluées en utilisant l'approche ascendante pour les biens d'équipement, le temps du personnel et les consommables. Les quantités de ressources ont été recueillies en interrogeant 60 professionnels de la santé dans 11 établissements de santé aux Philippines.

**CONCLUSION:**

Notre étude présente les premières estimations des coûts des services ambulatoires intégrés pour le traitement du DM et de soins de la TB dans un pays à revenu faible ou intermédiaire. Il est suggéré d'approfondir l'étude du rapport coût-efficacité et de l'accessibilité des services de détection de la DM chez les patients atteints de TB, compte tenu des coûts impliqués.

Diabetes mellitus (DM) causes a substantial financial burden for patients, their households and health systems, as well as disabilities and mortality.^1–3^ DM is known to increase the risk of progressing to active TB disease.^4–6^ The Philippines has a high TB incidence with comorbid DM.^7^ Estimated TB incidence in the Philippines was 638 per 100,000 population, and 22,000 adult TB incident cases were attributable to DM in 2022.^8^ DM not only increases the risk of developing TB but also has adverse effects on TB treatment outcomes, particularly if DM is not properly managed.^4–6^ Increasing early diagnosis and improving management of DM may accelerate the decline of TB incidence and unfavourable treatment outcomes thus contributing to ongoing transmission.^4,6,9^ Considering the disease burden of comorbid TB-DM, the WHO published a guideline to develop and implement collaborative actions aimed at reducing the double burden of TB and DM. The guideline included bi-directional screening of TB in people with DM, and of DM in people with TB.^10^ Despite increased political and public health awareness of DM management within TB services, there is a lack of evidence to promote policy for TB patients with comorbid DM. In the Philippines, the current TB National Strategic Plan includes integrating TB with other health programmes, including non-communicable diseases as a key activity, targeting 90% of TB cases to be screened for DM by 2022.^11,12^ This requires further understanding of the cost of providing DM screening and diagnostic services for TB patients, and the subsequent costs in providing routine DM services such as those incurred for drug prescriptions and the regular monitoring of blood sugar levels.

A recent systematic review for DM provider costs that included 52 publications investigated costs per outpatient visit and annual inpatient, laboratory and drug costs.^13–17^ None of these studies assessed costs of DM services for TB patients. In the Philippines, only one study assessed provider costs and the availability of DM services. However, the study is outdated and only assessed unit cost for DM medications.^18^ Other cost categories such as screening, human resources and equipment costs have not yet been investigated. The present study aimed to assess the costs of providing outpatient DM services that can be integrated into TB services.

## METHODS

### Scope, study sites, timing and population

We estimated the provider costs for delivering 11 services as part of an algorithm integrating DM services into TB services from the health system perspective (Supplementary Data S1), including risk assessment, screening and diagnosis, drug prescriptions and consultations. Unit costs of DM services were estimated using data collected from sites not currently integrating services but providing both services. We estimated unit costs excluding overhead and start-up costs. A cross-sectional study was conducted in health facilities that provide outpatient DM services in Negros Occidental, the Philippines. Health professionals who provide DM care were identified at each study site; we sought to conduct 5–6 interviews with key informants, including one medical doctor, two nurses or midwives, one laboratory technician and one staff in charge of procurement and/or finance at each site (Supplementary Table S2). A total of 60 respondents were purposively selected and enrolled in our study, which was a nested study within an ongoing cohort study assessing the effects of malnutrition on TB treatment outcomes in Manila, Negros Occidental and Cebu in the Philippines, and the study sites of our nested study were selected from the main cohort study.^19,20^ Other study sites in Manila and Cebu were not included in our study due to difficulty in data collection during COVID-19 disruptions. Data were collected between October 2021 and June 2022, and all the data were collected retrospectively for 2021.

### Data collection

The WHO *“Costing Guidelines for Tuberculosis Interventions”* was adapted for data collection, analysis and reporting to assess the cost of outpatient DM services in this study.^21–24^ Data were collected using a combination of interviews and timesheets. Data were also extracted from financial and administrative documents obtained from the finance and human resource departments in each health facility. Resource quantity data were obtained from interviews with healthcare professionals who worked in outpatient DM services. In addition, general information, such as facility size in square meters, the catchment population and service utilisation, including the number of outpatient visits, DM screening and diagnosis tests performed, and the number of people on DM management, was also obtained from the facility records. A bottom-up (BU) approach was adopted to collect unit cost of DM drugs, consumables and equipment using a semi-structured interview guide. In order to evaluate time spent by the staff, both the BU and top-down (TD) approaches were adopted using semi-structured interviews and staff timesheets (Supplementary Data S3).

Prior to interviews with healthcare professionals, we conducted an interview with the head of each health facility to obtain permission to conduct interviews and identify key informants who could provide necessary data and information on facility infrastructure, finance and human resources and types of outpatient DM services provided by the facility. Key informant interviews were performed to estimate resource utilisation and time consumed in the previous month, and staff timesheets were used to determine time spent from self-reported working schedule in a week.

Annual staff salaries were estimated from the monthly salary and allowances per staff collected from records of human resources (Supplementary Table S4). The value of the buildings at each study site was estimated using the size of the facility and the official government construction costs. The useful life of capital goods were assumed to be 30 years for buildings and 5 years for equipment.^25^ A 5% wastage rate was applied for medical consumables and drugs (Supplementary Table S5). All of the cost data were collected in Philippine pesos (PHP) and converted to US dollars (USD) using UN Operation Exchange Rate across the data collection period from October 2021 to June 2022 (1USD = PHP52.1).

### Data analysis

Collected data were entered, checked for reasonableness and cleaned using MS Excel (Microsoft, Redmond, WA, USA) for each study site, and then merged and imported into R Statistical Software v4.2.0 (R Computing, Vienna, Austria) for further analysis. Costs were calculated separately for consumables, drugs, equipment, building and staff for each DM service, and then combined to estimate the unit cost per DM service. Mean and standard deviation (SD) were used to summarise the unit cost per DM service. Results with median and interquartile ranges were summarised in the supplementary information (Supplementary Table S8–S10).

Cost per DM case detected among TB patients was calculated as the total costs of DM screening and diagnosis for a specified population using a particular screening and diagnostic algorithm divided by the total number of cases identified.^26^ The screening and diagnostic testing approaches and algorithms available for each study site were obtained from interviews with nurses and laboratory technicians providing those services at the respective facilities. The cost per DM case detected was then estimated for five different algorithms, combined with glycated haemoglobin (HbA1c) test, random plasma glucose (RPG), fasting blood sugar (FBS) and oral glucose tolerance test (OGTT). This included the diagnostic algorithms comprised of one stage (confirmatory test) or two stages (screening test and confirmatory test). Furthermore, results were categorised according to all age groups, with those aged over 45 years were identified as a high-risk population with DM.^27^

As there were no integrated DM services for TB patients at our study sites, the proportion of TB patients who tested positive for DM screening and confirmatory tests were extracted from the main cohort study (Supplementary Table S6).^19,20^ The participants of the main study were initially screened for DM using either HbA1c or RPG; those with an HbA1c >5.7% or RPG >11.1 mmol/L. OGTT was provided as a confirmatory test for DM. The sensitivity and specificity of each test and the estimated prevalence of DM were taken from the literature available as assumptions for calculating the positive predictive values of each test (Supplementary Table S6).^28–32^ The monthly cost of treating each patient was estimated using the cost data of outpatient DM services that are periodically performed for people with DM.

### Ethics considerations

The Asian Eye Institute Ethics Review Committee (Makati City, The Philippines) reviewed and provided the Philippines national ethics approval (2021-010). Approvals were also obtained from the London School of Hygiene & Tropical Medicine, London, UK (#25149) and Nagasaki University, Nagasaki, Japan (NU_TMGH_2021_153_1). Written consent was provided by all participants before the commencement of the interview. The informed consent signed by all participants explicitly stated that only the principal investigators could access the study dataset.

## RESULTS

### Facility characteristics

The total number of outpatient visits at the 11 study sites ranged from 1,376 to 4,782 in 2021; of these, outpatient visits for DM services accounted for 3% to 13% of total outpatient visits (Supplementary Table S2).

### Unit cost for diabetes care

Risk assessment, screening and diagnosis with FBS or RPG, drug prescription, consultation visits and referral services to other facilities for diagnostics and complications were the most commonly provided outpatient services during DM visits. Screening using the HbA1c test and diagnosis with OGTT were only provided in one private hospital. OGTT had the highest cost per patient, at USD23.72, while costs for risk assessment were only USD0.53 (SD ±0.20) (Table 1). Outpatient DM screening and monitoring were provided predominantly using FBS or RPG. Screening and diagnosis using FBS with chemistry analyser had a higher cost than that with glucometer (USD2.99, SD ±0.75 vs. USD1.67, SD ±0.83). Using the proportion of people taking each type of DM medicine in each facility, the weighted mean monthly drug cost per patient was estimated at USD7.67 per month. Staff was the main cost driver for outpatient DM visits where a laboratory testing was not required (i.e., consultation visits, drug prescriptions, risk assessments), ranging from 70% to 92%. Consumables were the main driver for screening and diagnosis services, ranging from 52% to 90% (Figure).

**Table 1.** Unit costs for DM interventions in sampled facilities in 2022 USD (USD1 = PHP52.1).

Intervention	Health facilities providing each type of DM outpatient services *n*	Cost breakdown by input type			Cost per patient Mean ± SD
		Consumable USD	Staff USD	Capital USD	
Risk assessment	10	0.10	0.37	0.06	0.53 ± 0.20
Screening and monitoring					
FBS: Glucometer	10	1.24	0.37	0.05	1.67 ± 0.83
RPG: Glucometer	11	1.00	0.28	0.04	1.32 ± 0.65
HbA1c	1	2.61	0.27	0.03	2.91
Diagnosis/confirmation					
FBS: Chemistry analyser	9	1.56	1.08	0.34	2.99 ± 0.75
RPG: Chemistry analyser	1	2.26	0.79	0.23	3.28
OGTT	1	19.63	3.57	0.51	23.72
Drug prescription	11	0.09	1.15	0.05	1.29 ± 0.43
Consultation visits	11	0.09	1.73	0.08	1.89 ± 0.84
Referral service					
General	10	0.08	0.64	0.06	0.78 ± 0.39
Complication	10	0.10	1.44	0.06	1.60 ± 0.67

DM = diabetes mellitus; USD = United States dollar; PHP = Philippine peso; SD = standard deviation; FBS = fasting blood glucose; RPG = random plasma glucose; HbA1c = glycated haemoglobin; OGTT = oral glucose tolerance test.

**Figure.**
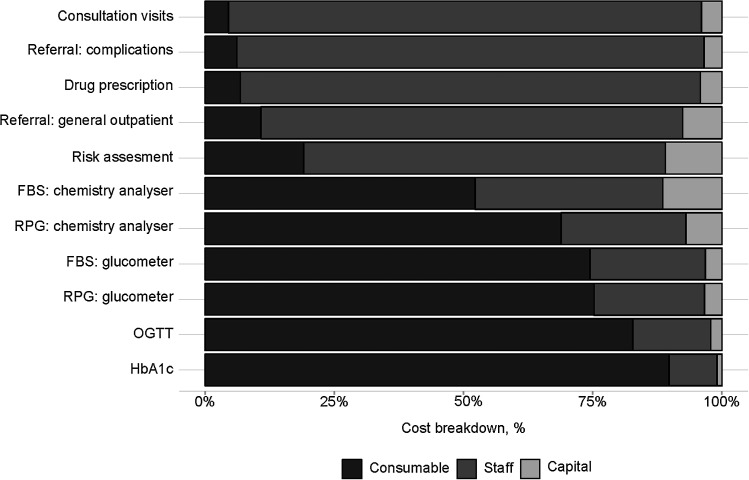
Cost drivers for providing diabetes outpatient services. Proportions are based on the mean unit cost per diabetes outpatient service. FBS = fasting blood glucose; RPG = random plasma glucose; OGTT = oral glucose tolerance test; HbA1c = glycated haemoglobin.

### Cost per DM case detected in patients with TB

Among the five diagnostic algorithms, one consisted of a screening blood test only, while the remaining four algorithms consisted of a screening blood test and a confirmatory blood test. The diagnostic algorithms with the lowest cost per case detected were 1) HbA1c, 2) screening by RPG and diagnosing by FBS, or 3) screening by HbA1c and diagnosing by FBS (Table 2).

**Table 2.** Estimated mean cost per case detected for diabetes among TB patients in 2022 USD (USD1 = PHP52.1).

Group	Algorithm		Cost per case detected
	Stage 1	Stage 2	Mean ± SD
All age groups			
	HbA1c >5.7%	—	17.80
	RPG >7.8 mmol/l*	OGTT	80.81
	RPG >7.8 mmol/l*	FBS >7.0 mmol/l^†^	17.43 ± 5.30
	HbA1c >5.7%	OGTT	78.72
	HbA1c >5.7%	FBS >7.0 mmol/l^†^	25.41
Age > 45 years			
	HbA1c >5.7%	—	10.62
	RPG >7.8 mmol/l*	OGTT	60.09
	RPG >7.8 mmol/l*	FBS >7.0 mmol/l^†^	11.73 ± 3.46
	HbA1c >5.7%	OGTT	58.80
	HbA1c >5.7%	FBS >7.0 mmol/l^†^	16.17

* Screening cost with glucometer was used for the estimation of cost per case detected.

^†^ Testing cost with chemistry analyser was used for the estimation of cost per case detected.

USD = United States dollar; PHP = Philippine peso; SD = standard deviation; HbA1c = glycated haemoglobin; RPG = random plasma glucose; OGTT = oral glucose tolerance test; FBS = fasting blood glucose.

The cost per case detected for these three algorithms ranged from USD17.43 (RPG + FBS) to USD25.41 (HbA1c + FBS). When the target population was limited to people aged over 45 years, the cost fell to USD11.73 (RPG+ FBS) to USD16.17 (HbA1c + FBS) per case detected.

### Unit cost for DM drugs

The types of DM medicines prescribed varied considerably across our study sites. Among oral medicines, metformin and gliclazide were most commonly prescribed. The mean monthly cost per patient for metformin (500 mg) was USD2.11 (SD ±0.75); for gliclazide, the mean monthly cost per patient ranged from USD2.92 (30 mg) to USD3.22 (80 mg), depending on the dose (Table 3).

**Table 3.** Mean unit costs for DM drugs in sampled facilities in 2022 USD (USD1 = PHP52.1).

Type of drug	Health facilities providing each type of DM medicine *n*	Monthly cost/patient Mean ± SD	Patients taking drugs %
Oral medicine			
Metformin (500 mg)	11	2.11 ± 0.75	95.6
Metformin hydrochloride + gliclazide (500 mg/80 mg)	1	10.08	0.1
Dapagliflozin/metformin hydrochloride 10/1,000 mg	1	36.99	5.3
Metformin-sitagliptin 100/1 mg	1	39.38	1.8
Gliclazide (80 mg)	3	3.22 ± 1.50	2.1
Gliclazide (60 mg)	2	2.80 ± 0.84	0.4
Gliclazide (30 mg)	5	2.92 ± 1.36	8.4
Glimepiride 4 mg	1	10.80	8.0
Glimepiride 3 mg	1	6.30	0.1
Glimepiride 2 mg	5	7.99 ± 0.48	3.7
Injectable medicine			
Biphasic isophane human insulin (Insuget 70/30)	7	29.45 ± 6.34	2.2
Scilin N 100 IU/ml	2	32.00 ± 11.00	0.1
Scilin M30 70/30	1	43.01	1.4
Regular insulin human u-100	1	33.52	0.1
Humulin 70/30	2	13.58 ± 9.62	0.3
Humulin I 100 IU/ml	4	15.75 ± 7.92	0.4

DM = diabetes mellitus; USD = United States dollar; PHP = Philippine peso; SD = standard deviation; IU = International Unit.

For injectable drugs, the brand available and its cost also varied by health facility. The mean monthly cost per patient ranged from USD13.58 to USD43.01. Biphasic isophane human insulin was most frequently prescribed, and the mean monthly cost per patient was USD29.45 (SD ±6.34).

### Monthly costs per outpatient

The total monthly costs per patient were estimated at USD8.95 (medicines and drug prescription only) to USD12.36 (medicines, drug prescription, monitoring and consultation visits) (Table 4).

**Table 4.** Estimated mean monthly costs per patient in 2022 USD (USD1 = PHP52.1).

Monthly services			Mean ± SD
DM medicine: drug prescription	—	—	8.95 ± 0.43
DM medicine: drug prescription	Monitoring by RPG (glucometer)	—	10.28 ± 0.84
DM medicine: drug prescription	Monitoring by FBS (glucometer)	—	10.47 ± 1.12
DM medicine: drug prescription	Monitoring by RPG (glucometer)	Consultation visits	12.17 ± 1.37
DM medicine: drug prescription	Monitoring by FBS (glucometer)	Consultation visits	12.36 ± 1.63

USD = United States dollar; PHP = Philippine peso; SD = standard deviation; RPG = random plasma glucose; FBS = fasting blood glucose.

## DISCUSSION

The aim of our study was to estimate the cost per DM case detected in TB patients in a low- and middle-income country, the Philippines. The cost per case detected with feasible and affordable algorithms (that is, using HbA1c, RPG and/or FBS) ranged from USD17.43 to USD25.41. The monthly outpatient cost per patient for DM drugs and outpatient services was estimated at USD8.95 to USD12.36. We provided the most recent evidence on the costs of providing outpatient DM services and the cost per DM case detected among TB patients in the Philippines. Our results will help with planning, budgeting and assessing the cost-effectiveness of providing integrated DM outpatient care within the TB programme.

A recent systematic review showed that the costs of DM outpatient services from the health system perspective, which were assessed using the same BU approach as our study, ranged from USD4–5 (Thailand, Brazil) to USD22 (Argentina) per outpatient visit, USD5 (Iran) to USD152 (Argentina) for annual laboratory costs, and USD26 (Thailand) to USD91 (Brazil) for drugs annually.^13–17^ Results of our study for outpatient visits and drugs were also in this range. In our study, the unit cost per outpatient DM service ranged from USD0.53 for risk assessment to USD23.72 for diagnosis using OGTT. Monthly drug cost per patient varied from USD2.11 to USD43.01.

In a previous study that assessed the costs of DM medicine in the Philippines in 2009, the monthly costs of oral and injectable medicines per patient was estimated at USD16, whereas this was USD7.67 in our study.^18^ The difference in the cost estimates could be due to the difference in study design and population. The previous study had interviews with people with DM and healthcare professionals in 30 hospitals across the capital city and four provinces. The study assessed costs of purchasing DM drugs from the patients’ perspective, and included private practitioners (e.g., private pharmacies). Costs may therefore have been overestimated due to the selling price, or the price difference between public and private service providers.

Our study has several limitations. First, the study was conducted in purposively selected public health facilities in one region in the Philippines, and is therefore only representative of settings in the public sector in rural areas in the Philippines. Second, the study did not include cost components such as overhead costs and training costs. In the VALUE-TB Study in the Philippines, which assessed provider costs for TB services, overhead cost was one of main contributors to TB services. The proportion of overheads costs was 40–50% of the cost of outpatient screening, monitoring, diagnostic and treatment visits.^21^ Therefore, we believe that estimated costs for DM services could also be higher if overhead costs are included. Third, information regarding the proportion of suspected DM cases and confirmed DM cases among TB patients was solely accessible through HbA1c measurements at 5.7% (for screening) and OGTT results (for diagnosis) as provided in the main cohort study. Therefore, our study had to assume the proportion of population screened as suspected DM with RPG (>7.8 mmol/l) and FBS (>7.0 mmol/l) in TB patients using the values from HbA1c (>5.7%) and OGTT. Furthermore, although DM risk assessment by scoring before blood tests is in practice in the Philippines, risk assessment data for TB patients were not available. We were therefore unable to include DM risk assessment in the estimation of cost per DM case detected in TB patients. Fourth, the scope of this study was to estimate the provider costs for outpatient DM services that can be integrated into TB care. For those applying our results, it is important to note that we did not look at costs of managing DM in the longer term (e.g., for DM-related complications or inpatient care), nor costs from the patient’s perspective,^33^ which both need to be considered when assessing affordability or cost-effectiveness.

## CONCLUSION

Our study provided the most recent data on the cost of providing outpatient DM services for TB patients. The results will help with planning, budgeting and assessing the cost-effectiveness of providing integrated outpatient DM care within the TB programme. Further studies are required to obtain more robust evidence around DM screening and diagnosis in TB patients, and to understand the costs of providing DM inpatient services and costs from the patient’s perspective.

## Supplementary Material


