# Supplementary material for: Health system costs of providing outpatient care for diabetes in people with TB in the Philippines
Source: IJTLD Open. 2024 Mar 1;1(3):124–9. doi: 10.5588/ijtldopen.23.0554 (PMC11221583; doi:10.5588/ijtldopen.23.0554)
Supplement: Supplementary file 1 [file iutld_ijtld_open_23.0554_supplementarydata1.pdf]

©2024 The Union http://dx.doi.org/10.5588/ijtldopen.23.0554

Health system costs of providing outpatient care for diabetes in people with TB in the Philippines

SUPPLEMENTARY DATA

Supplementary Data S1. Health system of TB and diabetes services in the Philippines

In the Philippines, costs for diabetes diagnosis and management are not fully covered by national insurance, the NTP or the non-communicable disease control programme, but direct medical costs for TB treatment and diagnosis are covered [1,2]. Social support is provided by the NTP for people with drug-resistant TB (DR-TB) with the purpose of improving treatment adherence and includes food packages and transportation fees for visiting health facilities [3-5]. Furthermore, The Department of Social Welfare and Development (DSWD) of the Philippines has a nationwide conditional cash transfer (CCT) programme for households living in poverty, and as of 2016, the CCT programme covered 4.4 million households, equivalent to 20% of the total population [6].

The current situation for the provision of TB and diabetes services differs by area and the level of facility. No diabetes outpatient services, except for drug pick-up at community level, are integrated within the TB programme. We identified two different patterns for providing TB and diabetes services in our study sites in the Philippines.

Pattern 1.

- Drug pick-up for TB & diabetes: provided at the community level (barangay health centre (BHC)).
  *BHC is classified as community level (Level 0 facility) for TB care in the Philippines [7].
- Diagnosis of and prescription for TB and diabetes: Not integrated. Provided at different levels of health facilities (diagnosis for TB is at city/rural health centres and diabetes is at district hospitals).
  *Urban/rural health centre including city health centre is classified as primary-care level (level 1 facility), and district hospitals as primary-care level with specialized health services (level 2 facility) for TB care in the Philippines [7].
- Diabetes screening and monitoring: 1st screening and regular monitoring by Point of Care (POC) HbA1c or fasting blood glucose are not provided in public health facilities, and therefore people living with diabetes have to visit private pharmacies.

Pattern 2.

- Drug pick-up for TB & diabetes: provided at the secondary health facility level (city or rural health unit). Some cities or rural health units do not allow TB medications to be provided at community level, and therefore, for TB drug pick-up, patients have to visit secondary health facility level where diabetes medications are also available. However, TB services and diabetes services are provided separately.
- Diagnosis of TB and diabetes: Not integrated. Provided at different levels of health facilities (diagnosis for TB is at city/rural health centres and diabetes is at district hospitals).
- Diabetes screening and monitoring: 1st screening and regular monitoring by Point of Care (POC) HbA1c or fasting blood glucose are not provided in public health facilities, and therefore people living with diabetes have to visit private pharmacies.

Supplementary Table S2.

Characteristics and outpatient visits at 11 sampled health facilities in Negros Occidental, the Philippines

Facility	Facility level	Ownership	Locality	Total outpatient visits (2021)	Outpatient visits for diabetes (2021)	Outpatient visits for TB (2021)	Diabetes diagnostic algorithms
**Bacolod Health Centre**	Health centre	Government	Rural	4,383	436	1,864	RPG+FBS
**Bago Health Centre**	Health centre	Government	Rural	3,184	274	1,263	RPG+FBS
**Bago Hospital**	Primary hospital	Government	Rural	1,609	N/A	N/A	RPG+FBS
**Don Salvador Rural Health Unit**	Health centre	Government	Rural	1,376	86	358	RPG+FBS
**Dr. Pablo O. Torre Memorial Hospital**	Tertiary hospital	Private	Rural	3,187	93	257	HbA1c, HbA1c+OGTT, HbA1c+FBS
**EB Magalona Rural Health Unit**	Health centre	Government	Rural	4,782	425	2,370	RPG+FBS
**La Carlota Health Centre**	Health centre	Government	Rural	2,333	180	1,021	RPG+FBS
**Pontevedra Rural Health Unit**	Health centre	Government	Rural	4,431	N/A	2,319	RPG+FBS
**San Enrique Rural Health Unit**	Health centre	Government	Rural	2,803	210	1,248	RPG+FBS
**Silay City Health Office**	Health centre	Government	Rural	3,621	176	1,620	RPG+FBS
**Valladolid Health Centre**	Health centre	Government	Rural	1,857	243	580	RPG+FBS

FBS: Fasting blood glucose, HbA1c: Glycated haemoglobin, OGTT: Oral glucose tolerance test, RPG: Random plasma glucose, N/A: Not available

Supplementary Data S3.

Methods for collecting and estimating unit costs

Bottom-up (BU) costing is an approach to estimate costs that involves detailed assessment and/or measurement of all materials and resources being used for providing health care services. The BU approach captures all the inputs utilized and consumed for a specific health service, by interviews, observations and/or reviewing work records/logs, then multiplies these input quantities by the unit cost of each input. Conversely, the top-down (TD) approach starts from total health expenditures/budgets, and the costs are estimated by allocating the total expenditures based on the utilization of each heath service [8].

Supplementary Table S4. Mean salary of healthcare workers involved in providing diabetes care in sampled facilities in the Philippines in 2022 USD (USD1 = PHP52.1)

Title	N*	Annual salary	
		Mean	SD
Medical doctor	18	18,362	2,845
Nurse	68	8,862	1,676
Midwife	94	5,456	382
Nursing assistant	2	3,754	N/A
Medical technologist	24	5,989	2,351
Pharmacist	1	7,512	N/A
Laboratory aid	3	2,427	153
Other (warehouse, inventory, purchasing)	9	3,514	1,065

* N is the number of healthcare professionals providing diabetes services across 11 health facilities.

USD: United States dollars, PHP: Philippine peso, SD: Standard deviation

Medical doctors or medical officers, nurses, midwives, and medical technologists were the healthcare professionals who most commonly provided diabetes outpatient services (**Table S4**). The mean estimated annual salary, including allowances, was USD 18,362 (SD 2,845) for medical doctors, USD 8,862 (SD 1,676) for nurses, USD 5,456 (SD 382) for midwives, and USD 5,989 (SD 2,351) for medical technologists. In three facilities, there were also auxiliary and administrative staff including nursing assistants (USD 3,754), pharmacists (USD 7,512), laboratory aids (USD 2,427), and procurement/inventory staff (USD 3,514).

Supplementary Table S5: Price sources and methods for estimating unit costs by input

Cost type	Assumed useful life (years)	Price source	Allocation method	Key assumptions
**Buildings**	30	Facility documents, government construction costs	Service statistics (outpatient visits)	
**Medical equipment**	5	Facility procurement price	Service statistics (outpatient visits)	
**Other equipment, furniture**	5	Facility procurement price	Service statistics (outpatient visits)	
**Clinical staff**	-	Human resource documents	Time sheet, interview	
**Support staff**	-	Human resource documents	Time sheet, interview	
**Medical supplies**	-	Facility procurement price	Service statistics (outpatient visits)	Wastage assumed 5%
**Other supplies**	-	Facility procurement price	Service statistics (outpatient visits)	Wastage assumed 5%
**Drugs**	-	Facility procurement price	Service statistics (outpatient visits)	
**Maintenance**	-		Not included	
**Utilities**	-		Not included	
**Transport**	-		Not included	
**Food**	-		Not included	
**Training**	-		Not included	

Supplementary Table S6. Assumptions used for estimating cost per case detected

Test	% exceeded cut-off		Prevalence of diabetes		Sensitivity	Specificity	Positive predictive value	
	All age	Age > 45	All age	Age > 45			All age	Age > 45
HbA1c > 5.7%	47.5%	59.8%	22.6%	32.0%	92.3%	48.5%	34.4%	45.8%
RPG > 7.8mmol/l	47.5%[1]	59.8%[1]	22.6%	32.0%	93.0%	59.0%	39.8%	51.6%
OGTT for those with HbA1c > 5.7% or RPG > 7.8mmol/l	39.0%	48.6%	22.6%	32.0%	100.0%	100.0%	100.0%	100.0%
FBS for those with HbA1c > 5.7% or RPG > 7.8mmol/l	39.0%[1]	48.6%[1]	22.6%	32.0%	56.0%	97.9%	88.6%	92.6%

[1] Since there are no available data of positivity rate with RPG > 7.8 mmol/l and FBS > 7.0 mmol/l in TB patients in the Philippines, the proportion with HbA1c > 5.7% and diagnosed as diabetes with OGTT in the main study was used for that of RPG > 7.8 mmol/l and FBS > 7.0 mmol/l respectively.

FBS: Fasting blood glucose, HbA1c: Glycated haemoglobin, OGTT: Oral glucose tolerance test, RPG: Random plasma glucose, mmol/l: millimoles per litre

Supplementary Table S7. Number of people screened and cases detected based on the assumptions in Table S6

Group	Algorithm		Number screened	Case detected
	Stage 1	Stage 2		
All age group	HbA1c > 5.7%	-	100,000	5,722
	RPG[2] > 7.8mmol/l	OGTT	100,000	18,003
	RPG[2] > 7.8mmol/l	FBS[1] > 7.0mmol/l	100,000	12,077
	HbA1c > 5.7%	OGTT	100,000	18,003
	HbA1c > 5.7%	FBS[1] > 7.0mmol/l	100,000	12,077
Age > 45	HbA1c > 5.7%	-	100,000	18,901
	RPG[2] > 7.8mmol/l	OGTT	100,000	29,063
	RPG[2] > 7.8mmol/l	FBS[1] > 7.0mmol/l	100,000	25,373
	HbA1c > 5.7%	OGTT	100,000	29,063
	HbA1c > 5.7%	FBS[1] > 7.0mmol/l	100,000	25,373

FBS: Fasting blood glucose, HbA1c: Glycated haemoglobin, OGTT: Oral glucose tolerance test, RPG: Random plasma glucose, mmol/l: millimoles per litre

Supplementary Table S8. Estimated median cost per case detected for diabetes among TB patients in 2022 USD (USD1 = PHP52.1)

Group	Algorithm		Cost per case detected	
	Stage 1	Stage 2	Median	Interquartile range
All age group	HbA1c > 5.7%	-	17.80	N/A
	RPG[2] > 7.8mmol/l	OGTT	80.81	N/A
	RPG[2] > 7.8mmol/l	FBS[1] > 7.0mmol/l	15.06	13.59-22.89
	HbA1c > 5.7%	OGTT	78.72	N/A
	HbA1c > 5.7%	FBS[1] > 7.0mmol/l	25.41	N/A
Age > 45	HbA1c > 5.7%	-	10.62	N/A
	RPG[2] > 7.8mmol/l	OGTT	60.09	N/A
	RPG[2] > 7.8mmol/l	FBS[1] > 7.0mmol/l	10.19	9.35-15.23
	HbA1c > 5.7%	OGTT	58.80	N/A
	HbA1c > 5.7%	FBS[1] > 7.0mmol/l	16.17	N/A

USD: United States dollar, PHP: Philippine peso, FBS: Fasting blood glucose, HbA1c: Glycated haemoglobin, OGTT: Oral glucose tolerance test, RPG: Random plasma glucose, mmol/l: millimoles per litre

Supplementary Table S9. Median unit costs for diabetes drugs in sampled facilities in 2022 USD (USD1 = PHP52.1)

Type	n	Monthly cost per patient	
		Median	Interquartile range
**Oral medicine**			
Metformin (500 mg)	11	2.24	1.86-2.52
Metformin HCL + Gliclazide (500mg / 80 mg)	1	10.08	-
Dapagliflozin/Metformin HCL 10/1000 mg	1	36.99	-
Metformin-sitagliptin 100/1mg	1	39.38	-
Gliclazide (80mg)	3	3.94	1.13-4.6
Gliclazide (60mg)	2	2.80	-
Gliclazide (30mg)	5	2.05	1.8-4.49
Glimepiride 4mg	1	10.80	-
Glimepiride 3mg	1	6.30	-
Glimepiride 2mg	5	8.19	7.5-8.38
Injectable medicine			
Biphasic Isophane Human Insulin (Insuget 70/30)	7	27.60	24.87-35.28
Scilin N 100IU/ml	2	32.00	-
Scilin M30 70/30	1	43.01	-
Regular Insulin Human u-100	1	33.52	-
Humulin 70/30	2	19.74	-
Humulin I 100 IU/ml	4	19.74	9.14-20.37

HCL: Hydrochloride, SD: Standard deviation, USD: United States Dollar, PHP: Philippine Peso, SD: standard deviation, IU/ml: International units per millilitre, mg: milligram.

Supplementary Table S10. Estimated median monthly costs per patient in 2022 USD (USD1 = PHP52.1)

Monthly services				Median	Interquartile range
Diabetes medicine	Drug prescription	-	-	8.79	8.71-9.39
Diabetes medicine	Drug prescription	Monitoring by RPG (glucometer)	-	10.14	9.67-10.99
Diabetes medicine	Drug prescription	Monitoring by FBS (glucometer)	-	10.22	9.67-11.77
Diabetes medicine	Drug prescription	Monitoring by RPG (glucometer)	Consultation	11.46	11.12-12.74
Diabetes medicine	Drug prescription	Monitoring by FBS (glucometer)	Consultation	11.46	10.99-14.29

FBS: Fasting blood glucose, RPG: Random plasma glucose, USD: United States Dollar, PHP: Philippine Peso

Supplementary Table S11. Unit costs for diabetes interventions by sampled facility in 2022 USD (USD1 = PHP52.1)

Study site	Risk assessment	HbA1c	Referral service		FBS		RPG		OGTT	Drug prescription	Consultation
			General	Complication	Glucometer	Chemistry analyser	Glucometer	Chemistry analyser			
Rural health unit #1	0.75		0.82	3.00	2.45	4.60	2.15			1.94	3.38
City health office #1	0.72		0.58	1.43	2.65		2.28			1.45	1.35
City health office #2	0.83		1.00	1.82	1.33	2.50	1.28			2.04	3.56
Rural health unit #2	0.33		0.60	1.02	0.91	3.02	0.90			1.10	1.31
City health office #3	0.63		0.67	0.72	0.84	2.88	0.75			1.72	0.75
Rural health unit #3	0.27		0.25	1.12	1.85	2.76	1.85			1.12	1.26
Rural health unit #4	0.27		0.65	0.94	1.52		1.50			0.65	1.57
Hospital #1						3.94	1.94			1.05	1.63
Hospital #2	0.51	2.91	0.50	2.06	3.32	2.42	0.15	3.28	23.72	1.24	2.06
Rural health unit #5	0.57		1.74	2.22	0.77	2.10	0.71			0.80	2.23
Rural health unit #6	0.39		1.02	1.66	1.04	2.66	1.05			1.05	1.70

FBS: Fasting blood glucose, HbA1c: Glycated haemoglobin, OGTT: Oral glucose tolerance test, RPG: Random plasma glucose, SD: Standard deviation USD: United States Dollar, PHP: Philippine Peso

Supplementary Data S12 Representativeness of our analysis

The subnational disease burden data for TB has not been fully assessed or understood in the Philippines. The latest TB prevalence survey was conducted in 2016, and the survey assessed TB prevalence at subnational level, by allocating 17 regions into four strata. The prevalence with bacteriologically confirmed TB was 1,159 (95% CI: 1.016-1,301) per 100,000 population, and it ranged at subnational level from 856 (95% CI: 686-1,026) in stratum 4 (Mindanao) to 1,358 (95% CI: 1,103-1,612) in stratum 1 (Metro Manila) per 100,000 population. The prevalence in stratum 2, which includes Negros Occidental Region where our study was conducted, was 1,234 (95% CI: 873-1,594) per 100,000 population [9], which is not significantly different from the national prevalence of TB.

The availability of the prevalence of diabetes among TB patients at subnational level is very limited in the Philippines, and only one study assessed the prevalence of comorbidities including diabetes among TB patients at TB diagnosis [10]. The study assessed the prevalence of diabetes among TB patients in three different areas of the Philippines (i.e. Metro Manila, Negros Occidental, and Cebu). The study showed that the overall prevalence of diabetes was 22.6% (N=881). Although Negros Occidental, where our study was conducted, had a high prevalence point estimate (25.5%) compared to the other two areas (20.1% and 20.9%), the study did not find a statistical significance (p=0.24). The overall prevalence (22.6%) was used for the analysis of cost per case detected in our analysis. As a sensitivity analysis, we assessed the range of cost per case detected when the prevalence of diabetes ranged from 20.1% to 25.5% for the algorithm with RPG (screening) + FBS (diagnosis). The cost per case ranged from USD 17.14 to USD 17.75 for all age group.

References for supplementary texts

1. Tan GH. Diabetes Care in the Philippines. *Ann Glob Health* 2015; **81**(6): 863-9.

2. Corporation PHI. Implementation of Primary Care Benefit 2 Package (PCB2 - outpatient medicines benefits for hypertension, diabetes, 2014.

3. National TB Control Programme Department of Health Philippines. Tuberculosis Financing in the Philippines, 2015.

4. Florentino JL, Arao RML, Garfin AMC, et al. Expansion of social protection is necessary towards zero catastrophic costs due to TB: The first national TB patient cost survey in the Philippines. *PLoS One* 2022; **17**(2): e0264689.

5. Department of Health. Philippines. National Tuberculosis Control Program Manual of Procedures 6th edition. Manila, Philippines, 2020.

6. The World Bank. FAQs about the Pantawid Pamilyang Pilipino Program (4Ps). 2017. https://www.worldbank.org/en/country/philippines/brief/faqs-about-the-pantawid-pamilyang-pilipino-program.

7. Garfin C, Mantala M, Yadav R, et al. Using Patient Pathway Analysis to Design Patient-centered Referral Networks for Diagnosis and Treatment of Tuberculosis: The Case of the Philippines. *J Infect Dis* 2017; **216**(suppl_7): S740-S7.

8. Cunnama L, Garcia Baena I, Laurence Y, et al. Costing guidelines for tuberculosis interventions. Geneva, Switzerland: World Health Organization, 2019.

9. Lansang MAD, Alejandria MM, Law I, et al. High TB burden and low notification rates in the Philippines: The 2016 national TB prevalence survey. *PLoS One* 2021; **16**(6): e0252240.

10. Cox SE, Edwards T, Faguer BN, et al. Patterns of non-communicable comorbidities at start of tuberculosis treatment in three regions of the Philippines: The St-ATT cohort. *PLOS Glob Public Health* 2021; **1**(11): e0000011.
